# Fully automated deep learning approach to dental development assessment in panoramic radiographs

**DOI:** 10.1186/s12903-024-04160-6

**Published:** 2024-04-06

**Authors:** Seung-Hwan Ong, Hyuntae Kim, Ji-Soo Song, Teo Jeon Shin, Hong-Keun Hyun, Ki-Taeg Jang, Young-Jae Kim

**Affiliations:** Department of Pediatric Dentistry, School of Dentistry, Seoul National University, 101 Daehak-ro, Jongno-gu, Seoul, 03080 Korea

**Keywords:** Dental development, Artificial intelligence, Deep learning, Demirjian method, Panoramic radiographs

## Abstract

**Background:**

Dental development assessment is an important factor in dental age estimation and dental maturity evaluation. This study aimed to develop and evaluate the performance of an automated dental development staging system based on Demirjian’s method using deep learning.

**Methods:**

The study included 5133 anonymous panoramic radiographs obtained from the Department of Pediatric Dentistry database at Seoul National University Dental Hospital between 2020 and 2021. The proposed methodology involves a three-step procedure for dental staging: detection, segmentation, and classification. The panoramic data were randomly divided into training and validating sets (8:2), and YOLOv5, U-Net, and EfficientNet were trained and employed for each stage. The models’ performance, along with the Grad-CAM analysis of EfficientNet, was evaluated.

**Results:**

The mean average precision (mAP) was 0.995 for detection, and the segmentation achieved an accuracy of 0.978. The classification performance showed F1 scores of 69.23, 80.67, 84.97, and 90.81 for the Incisor, Canine, Premolar, and Molar models, respectively. In the Grad-CAM analysis, the classification model focused on the apical portion of the developing tooth, a crucial feature for staging according to Demirjian’s method.

**Conclusions:**

These results indicate that the proposed deep learning approach for automated dental staging can serve as a supportive tool for dentists, facilitating rapid and objective dental age estimation and dental maturity evaluation.

## Background

Dental age estimation plays a significant role in forensic odontology in identifying individuals and in clinical applications to determine the degree of maturation in individuals [1, 2]. Children of the same chronological age may exhibit differences in the developmental stages of various biological systems, and dental age is one of the indices developed to assess a child’s developmental stage in a certain biological system [3]. In children with developing dentition, dental age is mostly assessed by tooth eruption or tooth development (calcification) [1, 4]. Because the exact time of tooth emergence is hard to determine and tooth eruption can be influenced by local exogenous factors, such as infection, lack of space, and premature extraction of deciduous teeth, evaluating tooth development using radiographs is considered a more accurate method for estimating a child’s dental age [3, 5]. Thus, dental development serves as a reliable indicator of biological maturity in children, as it is less affected by nutritional and endocrine factors [6]. It is mainly influenced by genes, whereas skeletal development is strongly affected not only by genes but also by nutrition and environmental factors [4].

Several methods for dental development assessment have been proposed, and one of the most widely used dental development staging systems is Demirjian’s method [6, 7]. Demirjian’s method evaluates the developmental stages of the seven teeth of the left mandible, except for the third molar, based on panoramic radiographs [8, 9]. Each tooth is divided into eight calcification stages, from stage A (beginning mineralization) to stage H (apex closed). The score of each stage is allocated, and the sum of the scores represents the subject’s dental maturity. The maturity score may be used to detect advanced or delayed dental maturity of the individual compared to reference subjects of the same age or be converted into dental age using available tables and percentile curves [2].

The use of radiographic methods for dental development assessment is a simple, quick, cost-effective, noninvasive, and reproducible technique that can be applied to determine the ages of both dead and living individuals [6, 7]. However, the limitation is that subjective scoring and the reproducibility of the operator’s measurement bias can influence the results [10]. Additionally, manual evaluation is time-consuming and may be complex in a disaster situation when a significant number of forensic identifications are needed [11–13]. With the advance of computer technology, artificial intelligence (AI) models have been introduced in forensic odontology to overcome these limitations and for more accurate diagnosis and support decision making [10, 11, 14, 15]. AI refers to a machine algorithm that is able to reason out and execute cognitive functions, and the two major subfields of AI are machine learning (ML) and deep learning (DL) [16, 17]. ML algorithms are closely related to computer statistics and are applied to enable machines to learn autonomously from data and perform specific tasks such as predictive analytics. DL is a subset of ML that imitates the architecture of biological neural networks in the brain based on artificial neural networks [17, 18]. DL consists of more than one hidden layer between the input and the output layers, organized in a deeply nested network architecture, which distinguishes it from simple artificial neural networks [19]. Numerous deep learning architectures, such as autoencoders, restricted Boltzmann machines (RBMs), deep belief networks (DBNs), convolutional neural networks (CNNs), recurrent neural networks (RNNs), distributed representations, and generative adversarial neural networks (GANs) have been applied in various areas. Among them, CNN-based methods have gained popularity in medical image analysis and are predominantly used in medicine and dentistry [19, 20]. AI technologies can be widely implemented in various fields of dentistry specialties, such as detecting dental caries, apical lesions, alveolar bone loss, osteoporosis, cancerous lesions, and predicting age estimation. With its high performance and increased efficiency, AI technology helps dentists improve the accuracy of diagnosis, develop preventive strategies, establish treatment plans, and predict treatment outcomes [18, 21].

AI technology has been applied to age estimation with various DL models and different age measurement methods using panoramic radiographs [14, 16, 18]. Wang et al. assessed two convolutional neural networks (CNNs), VGG16 and ResNet101, for dental age estimation, and the VGG16 model exhibited high accuracy in predicting age groups [10]. Guo et al. reported better performance of CNN models to age threshold classification than the manual method [22], and Kahaki et al. suggested that the deep learning model can efficiently classify the images with high performance that enables automated age estimation with high accuracy and precision [23].

However, most previous studies on using deep learning for age determination were mainly focused on classifying into ‘age groups’, which may be a broad spectrum for individual identification and show difficulties in utilizing it to clinical practice of accurate individual’s dental age or development. The studies applying AI technology to currently used dental age estimation methods for accuracy and efficiency are limited. To aid clinicians and forensic odontologists in utilizing dental age estimation methods with the advancement of AI technology, it is necessary to investigate whether current dental age estimation methods can be implemented using deep learning models and whether distinctions in the development of individual teeth can be well distinguished. Therefore, the present study aimed to develop and evaluate the performance of a fully automated deep learning approach for dental development assessment based on Demirjian’s staging system in panoramic radiographs.

## Materials and methods

### Dataset collection

The panoramic radiograph datasets used in this study were obtained retrospectively from the 2020–2021 database of the Department of Pediatric Dentistry at Seoul National University Dental Hospital. The subjects’ ages ranged from 4 to 16 years, and they were of Korean ethnicity. For the utilization of dental developmental staging with Demirjian’s method, panoramic images with low resolution, a subject’s pathologic condition affecting the maturity of teeth, missing permanent teeth in the left mandible, a history of orthodontic treatment, the existence of apical lesions and eruption disturbances of teeth were excluded from the study.

This study was conducted in accordance with the principles of the Declaration of Helsinki and was approved by the Institutional Review Board of the Seoul National University Dental Hospital, Seoul, Korea (Ethics Code: ERI23026). Informed consent was waived by the Ethics Committee of Seoul National University Dental Hospital for this retrospective study, as the data and patient details were anonymized.

### Proposed methodology

**Fig. 1:**
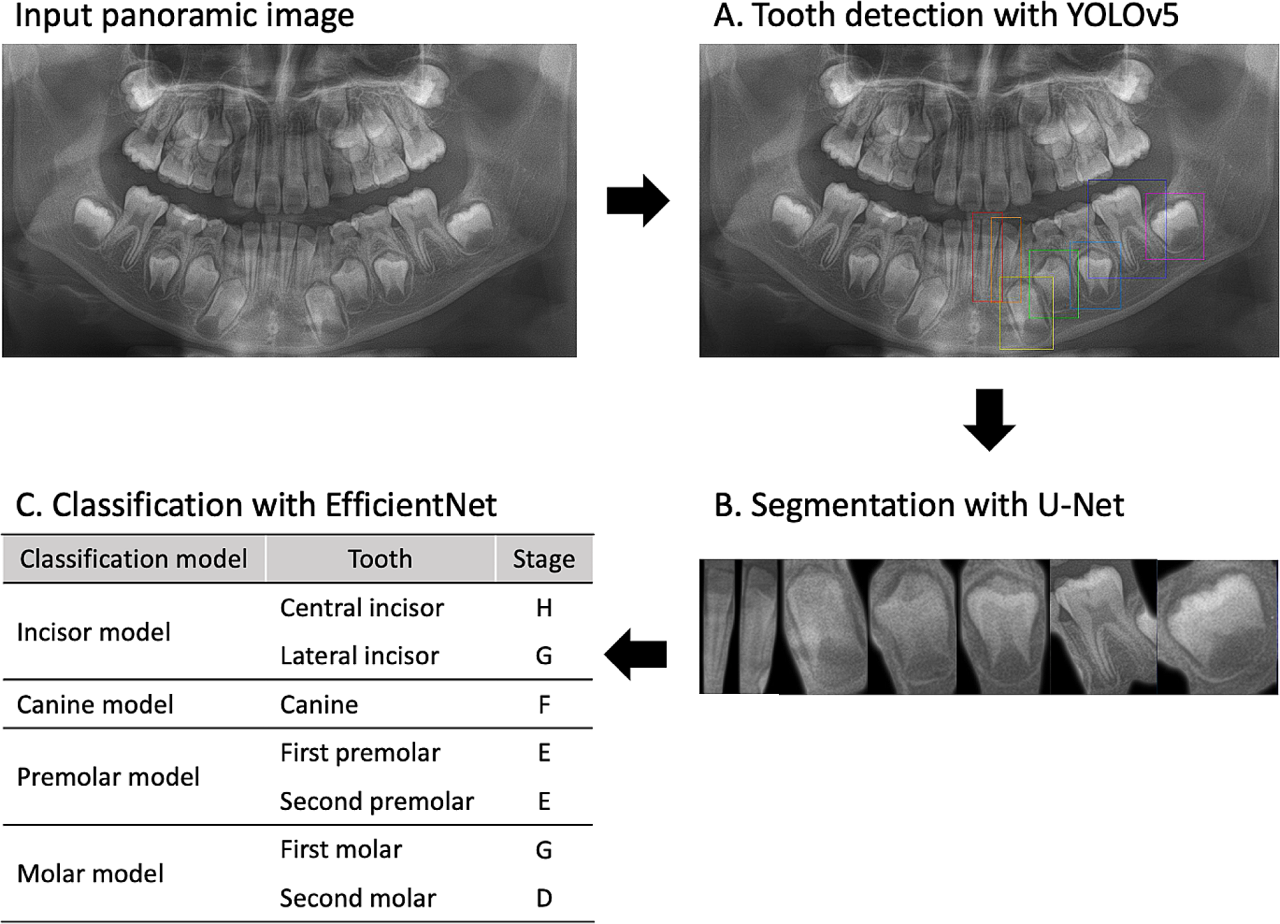
Workflow of the proposed fully automated dental development assessment system including three procedures: (**A**) Detection, (**B**) Segmentation, and (**C**) Classification

In this study, a novel approach for an automated dental development stage classification system based on panoramic images was proposed. The proposed methodology includes three key procedures using CNN models: detection, segmentation, and classification. First, the Yolov5 detection model automatically detected and individually cropped the seven permanent teeth of the left mandible in sequence, starting from the front. Second, the cropped images were processed with U-Net model to segment each tooth from its surrounding background. Finally, the segmented seven teeth were assigned to the EfficientNet classification model (Incisor, Canine, Premolar, and Molar) in sequence and classified into dental development stages based on Demirjian’s method. The performances of the models used in each procedure were analyzed. Figure 1 illustrates the workflow of our proposed methodology for a fully automatic dental developmental stage assessment system. Gradient-weighted class activation mapping (Grad-CAM) was employed to analyze the heatmap images of the model for each developmental stage.

### Tooth detection using YOLOv5

The You-Only-Look-Once (YOLO) v5 model was used for the detecting the seven permanent teeth of the left mandible. The YOLO system is a fast and accurate object detector model that uses a single neural network and predicts bounding boxes and class probabilities directly from full images in one evaluation [24]. The YOLO network consists of three main parts. Backbone: A pre-trained convolutional neural network used to extract feature representation for images. Neck: This part connects the backbone and the head, mixing and combining the features formed in the backbone. Head: Responsible for generating the final output. It applies anchor boxes on feature maps and renders the final output. The panoramic radiographs were resized to 1000 pixels in width and 500 pixels in height. 80% of the 5133 panoramic samples were randomly allocated as the training dataset, and the remaining 20% were allocated for the validation dataset. The seven teeth of the left mandible were manually annotated with bounding boxes and annotated as ‘target’, and the rest of the teeth were annotated as ‘no_target’. When both primary and subsequent permanent teeth were present, the primary teeth were annotated as ‘no_target’ to ensure that only the permanent teeth were recognized. The image size was set to 640 × 640 with YOLOv5, and the training images were rotated from − 30 to 30 degrees, and the brightness and contrast were randomly changed within 30%. The transfer learning technique with the YOLOv5l(large) pre-trained model was used to accelerate and improve the performance. Transfer learning is a useful way to quickly retrain a model on new data without having to retrain the entire network.

### Tooth segmentation using U-Net

Tooth segmentation was performed to extract accurate and distinctive features of teeth and improve the accuracy of the dental development classification model by removing the surrounding background of the tooth from the cropped image. The U-Net model was employed to segment teeth in cropped images obtained from the previous tooth detection stage. The U-Net architecture consists of a contracting path (left side) to capture context and a symmetric expanding path (right side) that enables precise localization [25]. The contracting path consisted of repeated applications of two convolutional layers with a kernel size of 3 × 3 and a stride of 1, each followed by a rectified linear unit (ReLU) and a max-pooling layer with a window size of 2 × 2 and a stride of 2 for down-sampling. The expansive path was composed of a repeated application of a transported convolutional layer with a kernel size of 2 × 2 and stride of 2 for up-sampling the feature map followed by concatenation with the corresponding feature map from the contracting path and two convolutional layers with a kernel size of 3 × 3 and stride of 1, each followed by a ReLU. The final convolutional layer with a kernel size of 1 × 1 and stride of 1 mapped a 64-component feature vector to the desired number of classes (tooth region: 1, other region: 0). U-Net has been widely used in biomedical segmentation applications, and its application to tooth segmentation in X-ray images has demonstrated superior results [26]. 80% of the cropped tooth images were randomly allocated as the training dataset, and the remaining 20% were allocated for the validation dataset. As the contour of the tooth is important for stage determination, and U-Net might not accurately segment the tooth edge details [27], our study intentionally extended the segmentation beyond the exact tooth contour. The image size is set to 128 × 128 with U-Net. To minimize unnecessary variance and improve the performance of the model, training images were rotated from − 15 to 15 degrees, and size changes within a 10% range were applied for augmentation.

### Dental development classification with EfficientNet

The EfficientNet model was employed to develop the dental development classification model. EfficientNets are a family of image classification models, and scaling methods that uniformly scales all dimensions of depth, width, and resolution using a compound coefficient. This compound scaling method enables easy scale up a baseline convolutional neural network to any target resource constraints in a more principled way while maintaining model efficiency [28]. EfficientNet-B0 is the base model, and EfficientNet-B1 to B7 have scaled variants of the base model. The transfer-learning with pre-trained EfficientNet-B7 was used to accelerate and improve the performance. Four types of classification models (incisor, canine, premolar, and molar) were devised, according to the Demirjian’s method for dental development staging. The seven cropped and segmented tooth images were assigned to the classification model in order. The first and second tooth images were assigned to the Incisor model, the third tooth image to the Canine model, the fourth and fifth tooth images to the Premolar model, and the sixth and seventh tooth images to the Molar model. Each image was then labeled with the corresponding tooth development stage. The Incisor and Canine models classify their corresponding teeth into stage C to H, while the Premolar and Molar models classify their respective teeth into stage A to H. The development stage for each segmented tooth image from the panoramic radiographs was labeled by one skilled pediatric dental specialist, and set as a reference for the classification model training and evaluation. The intraobserver reliability of the developmental stage labeling of each tooth based on the Demirjian’s method was assessed using weighted Cohen’s kappa analysis with MedCalc^®^ Statistical Software (version 20.100; MedCalc Software Ltd, Ostend, Belgium). The developmental stage of each tooth was re-examined using 200 randomly selected panoramic radiographs at 3-week intervals, and the calculated weighted Cohen’s kappa values were 0.93, indicating ‘almost perfect’ agreement. Due to significant variations in the number of images for each stage of tooth development within the model, the data for training and validation in each development stage were randomly allocated as an 80:20 ratio, and the maximum number of training data was set to prevent significant training bias between categories. The image size is set to 224 × 224 with EfficientNet, and various data augmentation techniques were performed to increase the amount of data and avoid overfitting and optimize the results. Training images were randomly flipped horizontally, brightness, contrast, saturation, and hue values were randomly changed within 30%, image movement and size changes within the 10% range, and random rotation within 360 degrees were applied.

### Model training options and evaluations

The study was performed on an NVIDIA Tesla K80 24 GB GPU, and Python, an open-source programming language (version 3.8.13; Python Software Foundation, Wilmington, DE, USA), using the PyTorch library (version 1.9.1), was used for the model development.

For the development of the automated tooth development staging system proposed in this study, a detection and segmentation procedure for the seven left mandibular teeth in panoramic radiographs was needed prior to the tooth classification. A total of 5133 panoramic images were randomly split into a training dataset (80%) and a validation dataset (20%), and YOLOv5 was trained for tooth detection. The training of the detection model with YOLOv5 uses the Adam optimizer with an initial learning rate of 1e-3 and a batch size of 4. The GIoU loss function was adopted, and the model was trained for 100 epochs, selecting the model with the best performance.

The performance of the detection model was evaluated with recall, precision, and mAP (mean average precision). The equations are shown in (1), (2), and (3)

1 $$mAP=\frac{1}{N }\sum _{k=1}^{k=n}{AP}_{k}$$

n: number of classes, AP: average precision.

The U-Net model was trained for the segmentation process. 80% of the cropped tooth images from the detection procedure were randomly split and assigned to the training dataset, while the remaining 20% were allocated to the validation dataset. For the training of the segmentation model with U-Net, the Adam optimizer and binary cross-entropy loss function were used, with an initial learning rate of 1e-4 and a batch size 10. The model was trained for 1000 epochs and the model with the best performance was selected. The segmentation model was evaluated for accuracy.

The classification procedure of the dental developmental stages was performed using EfficientNet, and four types of classification models were developed based on Demirjian’s method: the Incisor model (central and lateral incisors), Canine model (canine), Premolar model (first and second premolars), and Molar model (first and second molars). Segmented images from U-Net were labeled with the corresponding tooth development stage. For each development class, datasets were randomly split, with 80% allocated to the training dataset and 20% to the validation dataset. The classification model with EfficientNet was trained for 1000 epochs using the Adam optimizer, and the best model was selected. The initial learning rate was set to 1e-4, and the batch size was 10, with the cross-entropy loss function being employed. A performance matrix was constructed to summarize the performance of the classification models. The recall (classification accuracy), precision, and F1 score for each classification model were calculated using the validation dataset, as shown in Eq. (2) to (4).

2 $$Recall=\frac{TP}{TP+FN}$$

3 $$Precision=\frac{TP}{TP+FP}$$

4 $$F1 score=2 \times \frac{Recall\times Precision}{Recall+Precision}$$

TP: true positive, FP: false positive, FN: false negative.

## Results

A total of 5133 panoramic radiograph images, consisting of 2825 males and 2308 females were retrospectively collected from the database of the Department of Pediatric Dentistry at Seoul National University Dental Hospital between 2020 and 2021. The age and gender distributions are presented in Table 1, with chronologic age calculated by subtracting the date of birth from the date of the panoramic radiograph taken.

**Table 1:** Age and sex distribution of the panoramic radiograph samples

Chronologic age	Boys	Girls
4–4.99	175	144
5–5.99	286	201
6–6.99	366	272
7–7.99	365	316
8–8.99	343	287
9–9.99	359	293
10–10.99	311	276
11–11.99	217	191
12–12.99	174	133
13–13.99	111	106
14–14.99	74	53
15–15.99	44	36
Total	2825	2308

### Performance of the detection and segmentation model

The performance of the YOLOv5 model was as follows: recall: 0.991, precision: 0.994, and mAP: 0.995. Recall measures how well you find true positives (TP) out of all predictions (TP + FN), and precision measures how well you find true positives (TP) out of all positive predictions (TP + FP) [29]. The mean average precision (mAP) is a commonly used metric to analyze the performance of an object detection model. A high mAP indicates that the model is more precise and has higher recall. The process of tooth segmentation with YOLOv5 is shown in Fig. 1A.

The accuracy of U-Net was evaluated for the performance, with accuracy, sensitivity, and specificity values all showing the same value. This is because the results of U-Net segmentation and ground truth contain only two grayscale intensity values, 0 and 255 [26]. The accuracy of the U-Net segmentation model was found to be 0.978, and the visualized images resulting from the U-Net can be seen in Fig. 1B.

### Performance of the classification model

The confusion matrix with recall (classification accuracy), precision, and F1 score for each classification model with the validation dataset is presented in Tables 2 and 3. The confusion matrix depicts the summary of the prediction results of a classification model. The F1 score combines precision and recall into a single metric and provides a balanced evaluation of a model’s performance. The F1 score has a range between 0 and 1, with 1 indicating perfect precision and recall and 0 representing poor performance [29]. The processes of fully automated classification are shown in Fig. 1C.

The Incisor model exhibited the highest classification accuracy in stage H (99.22) and the lowest in stage C (34.78), with the highest F1 score achieved in stage H (96.49). The Canine model demonstrated the highest classification accuracy in stage F (94.04), the lowest in stage G (65.89), and the highest F1 score in stage F (91.09). The Premolar model showed the highest classification accuracy in stage F (92.28), the lowest in stage G (73.37), and the highest F1 score in stage F (92.28). Last, the Molar model showed the highest classification accuracy in stage B (96.49) and the lowest in stage A (82.35), with the highest F1 score in stage D (94.08). Among the four classification models, the Molar model exhibited the best performance with the highest classification accuracy (90.97) and F1 score (90.81), while the Incisor model showed the lowest accuracy (66.49) and lowest F1 score (69.23). Cross-tabulations of the stages assigned within the validation dataset, using the ground truth data labeled by one skilled pediatric dentist (rows) and the classification model (columns), are shown in Tables 4, 5 and 6, and 7. In cases of misclassification, most misclassified stages were seen only in the neighboring stages.

**Table 2:** Evaluation metrics of each classified stage in incisor and canine classification models using EfficientNet

Classified stages	Incisor			Canine		
	Recall (%)	Precision (%)	F1 score (%)	Recall (%)	Precision (%)	F1 score (%)
C	34.78	61.54	44.44	77.78	83.05	80.33
D	66.67	60.95	63.68	70.00	77.78	73.68
E	80.72	75.00	77.75	80.82	82.71	81.76
F	81.79	87.16	84.39	94.04	88.32	91.09
G	35.74	75.91	48.60	65.89	81.73	72.96
H	99.22	93.90	96.49	92.04	77.61	84.21
Average	66.49	75.74	69.23	80.09	81.87	80.67

**Table 3:** Evaluation metrics of each classified stage in premolar and molar classification models using EfficientNet

Classified stages	Premolar			Molar			
	Recall (%)	Precision (%)	F1 score (%)	Recall (%)	Precision (%)	F1 score (%)	
A	80.00	88.89	84.21	82.35	100.00	90.32	
B	89.74	89.74	89.74	96.49	85.94	90.91	
C	87.96	88.79	88.37	91.41	90.85	91.13	
D	84.16	84.16	84.16	92.98	95.21	94.08	
E	87.05	87.52	87.29	92.70	94.07	93.38	
F	92.28	92.28	92.28	90.51	90.73	90.62	
G	73.37	69.86	71.57	85.58	79.26	82.30	
H	81.18	83.13	82.14	92.12	95.45	93.76	
Average	84.47	85.55	84.97	90.52	91.44	90.81	

**Table 4:** Cross-tabulation of the classified stages of the incisor assigned by the expert (column) and by the automated staging proposed method (row) (%)

Stages	C	D	E	F	G	H
C	**0.35**	0.65				
D	0.05	**0.67**	0.28			
E		0.11	**0.81**	0.07		
F			0.09	**0.82**	0.05	0.04
G				0.07	**0.36**	0.57
H					0.01	**0.99**

**Table 5:** Cross-tabulation of the classified stages of the canine assigned by the expert (column) and by the automated staging proposed method (row) (%)

Stages	C	D	E	F	G	H
C	**0.78**	0.21	0.01			
D	0.08	**0.70**	0.22			
E		0.05	**0.81**	0.14		
F			0.02	**0.94**	0.03	0.01
G				0.12	**0.66**	0.22
H				0.01	0.07	**0.92**

**Table 6:** Cross-tabulation of the classified stages of the premolar assigned by the expert (column) and by the automated staging proposed method (row) (%)

Stages	A	B	C	D	E	F	G	H
A	**0.80**	0.20						
B	0.02	**0.90**	0.08					
C		0.01	**0.88**	0.11				
D			0.07	**0.84**	0.09			
E				0.05	**0.87**	0.08		
F					0.04	**0.92**	0.04	
G						0.13	**0.73**	0.14
H						0.01	0.18	**0.81**

**Table 7:** Cross-tabulation of the classified stages of the molar assigned by the expert (column) and by the automated staging proposed method (row) (%)

Stages	A	B	C	D	E	F	G	H
A	**0.82**	0.18						
B		**0.97**	0.03					
C		0.04	**0.91**	0.05				
D			0.04	**0.93**	0.03			
E				0.02	**0.93**	0.05		
F					0.02	**0.91**	0.07	
G						0.07	**0.86**	0.07
H							0.08	**0.92**

### Visualization of Grad-CAM for the classification model

**Fig. 2:**
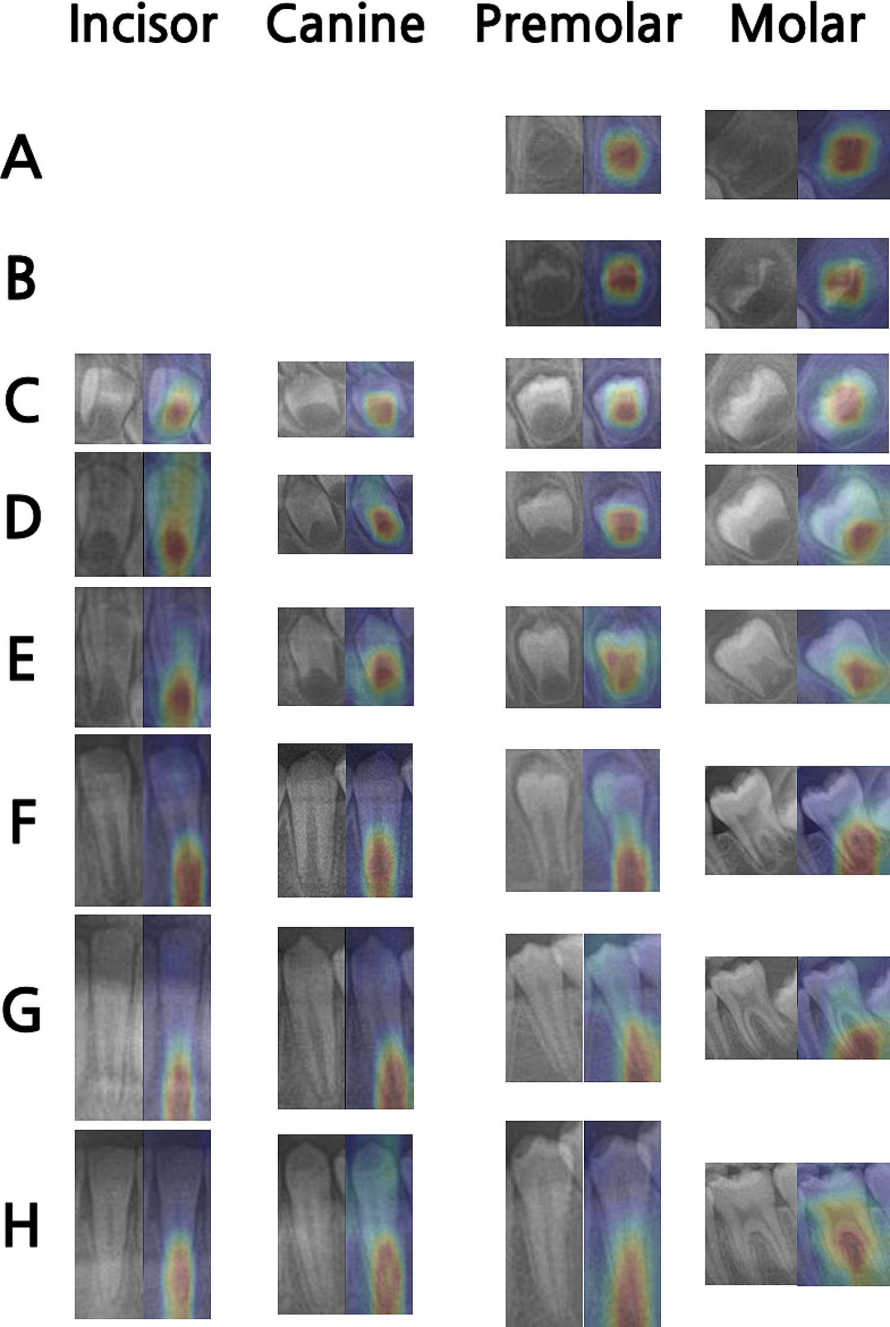
Grad-CAM heatmaps of the classification according to dental development stage by Demirjian’s method

Gradient-weighted class activation mapping (Grad-CAM) was applied to the classification model results to create a visual explanation of the regions on which the EfficientNet model concentrated for each tooth developmental stage. The areas that had the most influence on the classification evaluation of the model are highlighted and presented as a heatmap [30]. Figure 2 illustrates the Grad-CAM heatmaps for each dental development stage. The classification model seemed to effectively focus on the features of each stage, mostly concentrating on the apical portion of the tooth.

## Discussion

With the advancement of AI technology, there has been an increased interest in its application to dentistry. AI models serve as supportive tools, providing more precise, rapid, and consistent diagnoses while enhancing the accuracy of prognostic predictions, particularly in the analysis and diagnosis of radiographic images [16, 21, 31]. In forensic odontology, the estimation of age groups using AI has shown promising results, with high accuracy and precision [14, 15]. However, the studies on the developing dentition of adolescents and children were insufficient. The present study devised an automated dental developmental staging system in panoramic radiograph using deep learning models and evaluated the performances for each process. The proposed methodology has potential applications in estimating dental age for forensic odontology and in treatment planning for orthodontics and pediatric dentistry, by providing dental professionals with the ease and efficiency of dental staging.

Previous studies utilizing deep learning to classify dental development stages with panoramic radiographs have primarily focused on evaluating one or two teeth rather than the lower left quadrant teeth commonly examined in traditional methods [15, 32]. Mohammad et al. assessed the left mandibular first and second permanent premolars from stage C to H with a deep learning model [12], and Merdietio Boedi et al. devised an automated tooth developmental staging system for the segmented left mandibular third molar [33]. However, determining dental age based on the development stage of a single or a few teeth may result in a broad age range. A comprehensive evaluation of multiple teeth, similar to the currently used manual methods, would enhance the accuracy and practical utility of age determination. In this study, we designed a fully automated dental development classification system using deep learning based on Demirjian’s method and evaluated the performance of the stage classification. Our proposed method comprises three stages: detection, segmentation, and classification, with the aim of automatically classify the dental development stages in panoramic radiographs.

For the classification of individual teeth, it was necessary to detect each tooth sequentially. YOLO, a fast real-time object detection model known for its high mean average precision, was utilized to detect permanent teeth in panoramic radiographs. YOLOv4 has previously demonstrated high performance in detecting permanent tooth germs on panoramic radiographs [34] and has also shown accurate and fast performance for automated tooth detection and numbering in panoramic radiographs [35]. In this study, the performance of YOLOv5 showed promising results, demonstrating high recall, precision, and mean average precision for the detection of permanent tooth in the lower left quadrant of panoramic radiographs. However, since only panoramic samples with all seven teeth intact were included for training and evaluation, excluding images of missing or supernumerary teeth, the model’s detection performance may have shown higher values.

The segmentation procedure was conducted after detecting the seven teeth with the bounding boxes. Segmenting the tooth from the surrounding background can enhance the stage classification performance of the model, as the remaining surrounding tissues may obscure correct stage allocation [33]. U-Net, known for its high performance in segmenting teeth in panoramic and periapical images, as well as different features of teeth in periapical images [26, 27, 36], was employed to segment detected teeth in this study, achieving a high accuracy of 0.978. For tooth development staging, Merfietio Boedi et al. suggested the full tooth segmentation type, which includes only the developing tooth structure [33]. However, in this study, rough segmentation with the surrounding pixels was implemented to reduce misclassification caused by the under-segmentation of the tooth edge [12, 26], as the obscurity of the boundary between the tooth root and alveolar bone may be a critical issue in tooth segmentation [27]. Since Demirjian’s method classifies teeth based on the apical portion of the developing tooth, it was necessary to prevent inadvertent cutting of the tooth and minimize background interference as much as possible.

Following detection and segmentation, each tooth was categorized into four types (incisor, canine, premolar, and molar) based on its tooth number. Subsequently, four separate models were trained using EfficientNet, each corresponding to one of these categories and referencing the dental development stage according to Demirjian’s method. The EfficientNet model family is smaller and faster than other previous models with its compound scaling techniques [28] and has shown promising results in the classification of dental images [37, 38]. The model’s performance in distinguishing between each developmental stage of the tooth was assessed, with the F1 score, precision, and classification accuracy (recall) of the four models being highest in the Molar model, followed by the Premolar, Canine, and Incisor models (Tables 2 and 3).

The Incisor model effectively distinguished developmental stages, particularly in the E, F, and H stages. However, the overall model performance was poor due to low classification accuracy in the C, D, and G stages, resulting in an F1 score of 69%. The low F1 score of the C and D stages in the Incisor model can be attributed to the limited number of panoramic radiograph samples in young children, leading to underfitting of the model caused by the insufficient number of samples. Moreover, stages C and D often overlap with primary teeth or appear rotated on radiographs, making it challenging for the model to accurately learn and distinguish these stages. In stage G, a considerable number of cases were misclassified as stage H, contributing to low accuracy (Table 4). The blurred, shortened, or unclear perspective of the lower incisors in panoramic radiographs with mixed dentition, which could result from improper positioning of the patient [39], may also attribute to the low performance of the Incisor model. Positioning errors are a common issue in panoramic radiography, causing image distortions where the apexes of the lower incisors may appear out of focus, impacting diagnostic accuracy [40]. Such errors are more prevalent among younger individuals who may not remain calm and motionless during the radiograph procedure, leading to challenges in proper positioning [39].

The Canine model exhibited higher classification performance than the Incisor model, with no significant differences between stages and an average F1 score of 80%. However, similar to the Incisor model, the classification accuracy was low in stage G and was often misclassified as stage H (Table 5). The Premolar and Molar models demonstrated the highest performance in distinguishing developmental stages overall, with average F1 scores of 85% and 90%, respectively (Table 3). The highest F1 score was observed in the F stage for the Premolar model and the D stage for the Molar model. The performance between stages did not exhibit substantial differences in either model. However, both the Premolar and Molar models, showed the lowest F1 score in the G stage and misclassified cases were assigned to the E and H stages in a similar proportion.

The important features for dental developmental stages in classification models were highlighted through heatmaps using gradient-weighted class activation mapping (Grad-CAM) in Fig. 2 to improve the interpretability of the classification model. The classification models specifically focused on the apical portion of the developing tooth, which is considered an important feature in distinguishing between the stages based on Demirjian’s method.

In this study, we proposed a three-step procedure for the automated classification of dental development stages in panoramic radiographs using deep learning. Preceding the classification, tooth detection and segmentation would enhance the overall performance of stage classification compared to the classification procedure alone. While deep learning models have demonstrated high accuracy in tooth detection and segmentation [26, 27, 35, 36], their performance for dental developmental stage classification remains insufficient. Previous studies on deep learning models for development stage classification have primarily focused on premolars or molars [12, 32, 33], with research on incisors and canines lacking. Therefore, the results of this study could provide ideas for further research in devising more accurate classification models for a comprehensive automated dental age and maturity analysis. The four types of classification models exhibited differences in accuracy and performance, with the Incisor and Canine models showing lower performance than the Premolar and Molar models. It remains challenging to classify all seven lower left teeth individually using deep learning without manual interpretation to estimate dental age or evaluate dental maturity according to Demirjian’s method. Manual intervention is still necessary to minimize errors from the deep learning model, and completely relying on decisions from deep learning models is insufficient. However, considering that the misclassified cases were predominantly categorized into neighboring stages (Tables 4, 5 and 6, and 7), it suggests that the deep learning models can effectively play a supportive role in classifying tooth development stages.

The use of deep learning in radiograph analysis can reduce observer fatigue and bias, handle large samples in a short amount of time, thus shortening the time of diagnosis and increases the efficiency of clinicians [14, 21, 33]. In contrast to manual interpretation, disagreements between observers are eliminated, and the results are independent of the skills or experiences of the observers. Furthermore, with ongoing technology advancements, new CNN architectures are continually being developed, leading to a gradual improvement in the performance of deep learning models. This enhanced performance is expected to further increase their effectiveness and broaden their application in medical image analysis in the future [41, 42].

There are still a few limitations to this study. First, panoramic radiographs with low resolution or showing patient positioning errors were included as long as they could be distinguishable by a pediatric dental specialist. This inclusion criterion may have resulted in a particularly lower performance of the anterior tooth model, as these errors are more common in pediatric patients. Further studies considering positioning errors in panoramic radiographs is necessary to enhance the model’s performance, particularly for anterior teeth. Second, as four classification models were trained with seven teeth from the same panoramic samples, the number of datasets varied for each tooth stage. The imbalanced datasets between the developmental stages may introduce bias in the classification model, necessitating additional research to address class imbalances in developing dentition. Third, the number of samples for early developmental stages was limited, as panoramic radiographs are not routinely taken at a young age. Studies with a larger number of samples for early developmental stages are needed to improve the model’s performance for this phase. Furthermore, with the advancement of deep learning models, additional studies would be needed to investigate the potential for achieving more precise and accurate detection, segmentation, and classification performances, as demonstrated in this study.

## Conclusion

In this study, we proposed a fully automated dental development staging system based on Demirjian’s method using deep learning. The proposed method consists of three stages: detection, segmentation, and classification. YOLOv5, U-Net, and EfficientNet were employed for each stage, and the models’ performance was evaluated, demonstrating good results across various metrics. The detection and segmentation procedures yielded promising results, with a mAP of 0.995 for the detection model and an accuracy of 0.978 for the segmentation model. The classification model demonstrated F1 scores of 69.23, 80.67, 84.97, and 90.81 for the Incisor, Canine, Premolar, and Molar models, respectively. In the Grad-CAM analysis, the classification model focused on the apical portion of the developing tooth, a crucial feature for staging according to Demirjian’s method. Further studies are needed to enhance the model’s performance for dental staging accuracy in anterior teeth. The proposed method holds great promise for future use in forensic odontology and clinical practice, serving as a supportive tool for the rapid and objective evaluation of dental age estimation and dental maturity.

## Data Availability

The data that support the findings of this study are available from the corresponding author, upon reasonable request.

## Abbreviations

AI: Artificial intelligence
ML: Machine learning
DL: Deep learning
CNN: Convolutional neural network
YOLO: You only look once
ReLU: Rectified linear unit
mAP: mean average precision
Grad-CAM: Gradient-weighted class activation mapping
